# Neutralizing Antibody Responses After Severe Acute Respiratory Syndrome Coronavirus 2 BA.2 and BA.2.12.1 Infection Do Not Neutralize BA.4 and BA.5 and Can Be Blunted by Nirmatrelvir/Ritonavir Treatment

**DOI:** 10.1093/ofid/ofad154

**Published:** 2023-03-21

**Authors:** Aaron F Carlin, Alex E Clark, Aaron F Garretson, William Bray, Magali Porrachia, AsherLev T Santos, Tariq M Rana, Antoine Chaillon, Davey M Smith

**Affiliations:** School of Medicine, University of California San Diego, La Jolla, California, USA; School of Medicine, University of California San Diego, La Jolla, California, USA; School of Medicine, University of California San Diego, La Jolla, California, USA; School of Medicine, University of California San Diego, La Jolla, California, USA; School of Medicine, University of California San Diego, La Jolla, California, USA; Department of Public Health, College of Education, Health and Human Services, California State University San Marcos, San Marcos, California, USA; School of Medicine, University of California San Diego, La Jolla, California, USA; School of Medicine, University of California San Diego, La Jolla, California, USA; School of Medicine, University of California San Diego, La Jolla, California, USA; VA San Diego Healthcare System, San Diego, California, USA

**Keywords:** COVID-19, neutralizing antibodies, reinfection, nirmatrelvir/ritonavir, Omicron

## Abstract

The factors contributing to the rapid emergence of severe acute respiratory syndrome coronavirus 2 (SARS-CoV-2) BA.4 and BA.5 subvariants in populations that experienced recent surges of BA.2 and BA.2.12.1 infections are not understood. Neutralizing antibodies (NAbs) are likely to protect against severe disease if present in sufficient quantity. We found that after BA.2 or BA.2.12.1 infection, NAb responses were largely cross-neutralizing but were much less effective against BA.5. In addition, individuals who were infected and treated early with nirmatrelvir/ritonavir (Paxlovid) had lower NAb levels than untreated individuals.

## Teaser

Factors contributing to COVID-19 reinfection following Omicron infection are not well understood. We found that neutralizing antibodies elicited by BA.2 or BA.2.12.1 infections were less effective against BA.5 and that early nirmatrelvir/ritonavir treatment was associated with lower neutralizing antibody levels.

Severe acute respiratory syndrome coronavirus 2 (SARS-CoV-2) Omicron (B.1.1.529) subvariants have caused successive waves with BA.5 recently replacing BA.2.12.1 as the dominant strain in the United States. Escape mutations in the spike protein of BA.2.12.1, L452Q and S704L, and BA.5, L452R, and F486V, evade neutralizing antibodies (NAbs) responses generated by vaccination or previous BA.1 infection [1–5]. Given that BA.2.12.1 and BA.5 both contain escape mutations at position L452, it is unknown whether BA.2.12.1 infections elicit cross-protection against BA.4 and BA.5.

Viral antigen exposure is likely an important determinant of antibody responses towards SARS-CoV-2 [6–8]. The oral protease inhibitor, nirmatrelvir, combined with ritonavir (NM/r) (Paxlovid) reduces viral shedding and progression to severe coronavirus disease 2019 (COVID-19) [9]. By reducing viral replication, NM/r could reduce viral antigen exposure, thus limiting NAb responses and perhaps leaving people susceptible to reinfection.

## METHODS

### Patient Consent Statement

All participants provided informed consent. Nasopharyngeal swabs and plasma samples from participants were obtained under protocols approved by the University of California San Diego (UCSD) Institutional Review Board (IRB) (Protocol Numbers 200236X and 181624).

### Severe Acute Respiratory Syndrome Coronavirus 2 Sequencing and Sequence Analysis

Severe acute respiratory syndrome coronavirus 2 full-genome sequencing was performed using the COVID-19 ARTIC v4 Illumina library construction and sequencing protocol (https://github.com/CDCgov/SARS-CoV-2_Sequencing). Amplicons were generated with the NEBNext VarSkip VSS2b Primer kit (https://github.com/nebiolabs/VarSkip) coupled with VarSkip Short v2 Supplemental Primers to address low coverage regions caused by BA.2 variants. Polymerase chain reaction conditions were 98°C for 30 seconds, followed by 35 cycles of 95°C for 15 seconds and 63°C for 5 minutes. Libraries were generated with the NEBNext ARTIC SARS-CoV-2 FS Library prep kit (Illumina) with NEBNext Multiplex Oligos for Illumina. Samples were sequenced using a 2 × 75 base pair paired-end reads. Reads were processed with the CLC Genomics Workbench V22 (QIAGEN). In brief, the workflow identifies individual SARS-CoV-2 sample variants by first trimming and mapping high-quality reads (>20) to the reference genome and then calling variants to generate a full-genome consensus for each sample. All consensus sequences were assigned to lineages by Pangolin (PMID: 34527285). Sequences were further aligned to a set of representative SARS-CoV-2 variants using NextAlign (PMID: 29790939). Amino acid variations across all coding regions of the sample isolates compared with the reference strain BA.2 were interrogated.

### Cells and Chemicals

TMPRSS2-VeroE6 cells (Sekisui XenoTech) were maintained in Dulbecco’s modified Eagle’s medium (DMEM) (Corning, number 10013CV) plus 10% fetal bovine serum (FBS) (BioWest), 1 × penicillin/streptomycin (Gibco, number 15140-122), and 1 mg/mL geneticin (Gibco, number 10131-027) at 37°C and 5% CO_2_.

### Severe Acute Respiratory Syndrome Coronavirus 2 Isolation and Propagation

All work with SARS-CoV-2 was conducted in biosafety level-3 conditions at the UCSD following the guidelines approved by the Institutional Biosafety Committee. Viruses from clinical samples were isolated at UCSD under IRB number 160524 (BA.2.3 and BA.5) and number 200236X (BA.2.12.1). Viruses were isolated on TMPRSS2-VeroE6 (BA.2.12.1 and BA.5) or on Calu3 cells (BA.2.3) followed by passage through TMPRSS2-VeroE6. Variants BA.2.12.1 and BA.5 were isolated from nasopharyngeal swabs stored in viral transport medium (VTM) at −80°C. Approximately 500 µL of material was diluted in DMEM + 2 × Antibiotic/Antimycotic (anti/anti) (Gibco, catalog number 15240-062) and added to confluent T25 flasks of TMPRSS2-VeroE6 cells. After 2 hours, inoculum was removed and replaced with DMEM + 2% FBS with 1 × anti/anti. Media was replaced the next day. When cytopathic effect (CPE) became apparent, viral supernatant was centrifuged at 1000 *×g* for 5 minutes at 4°C, and aliquots were stored at −80°C. For BA.2 experiments, we utilized a BA.2.3 clinical isolate with identical spike protein to the BA.2 virus sequenced from nasopharyngeal swabs taken from the cohort infected with BA.2. BA.2.3 was isolated on Calu3 cells from a nasopharyngeal swab stored in VTM at −80°C. Sample was serially diluted in minimal essential medium (MEM) + 1 × anti/anti and incubated on cells. Input was removed and replaced with MEM + 2% FBS, 1 × GlutaMAX, 1 × sodium pyruvate, and 1 × anti/anti. When CPE appeared, BA.2.3 was further passaged once through TMPRSS2-VeroE6 so that neutralization assays were performed using viruses grown on the same cell type. All stocks were verified by deep sequencing.

### Authentic Severe Acute Respiratory Syndrome Coronavirus 2 Neutralizing Antibody Assay

Neutralization was determined by focus reduction neutralization test. Plasma samples were heat-inactivated at 56°C for 30 minutes, centrifuged at 8000 rpm for 5 minutes, and then aliquoted and frozen at −80°C until use. Four-fold serial dilutions of plasma samples in DMEM + 1% FBS were incubated with 100–250 focus-forming units of authentic SARS-CoV-2 diluted in DMEM for 1 hour at 37°C. Confluent TMPRSS2-VeroE6 cells in 96-well plates were washed once with PBS then infected with the virus + antibody mixture for 1 hour with gentle rocking. Inputs were removed, and cells were overlaid with 1% methylcellulose in MEM + 2% FBS and incubated for 24 hours at 37°C. Cells were then fixed with 4.5% formaldehyde for at least 30 minutes and stained with anti-SARS-CoV-2 nucleocapsid primary antibody (GeneTex, gtx135357) and anti-rabbit AlexaFluor 594 secondary antibody (Thermo Fisher Scientific) with Sytox Green nuclear counterstain. Whole-well images were acquired on an Incucyte S3 imager. Foci were counted using the Incucyte software, and percentage neutralization was calculated relative to media-only control wells on each plate. The SARS-CoV-2 neutralization titers were defined as the sample dilution at which a 50% reduction (NT50) in foci was observed relative to the average of the virus control wells. The geometric mean NT50 was calculated from at least 2 independent experiments each done using 2 biological replicates. Best-fit curves determining NT50 were generated in GraphPad Prism 9.

## RESULTS

**Figure 1.**
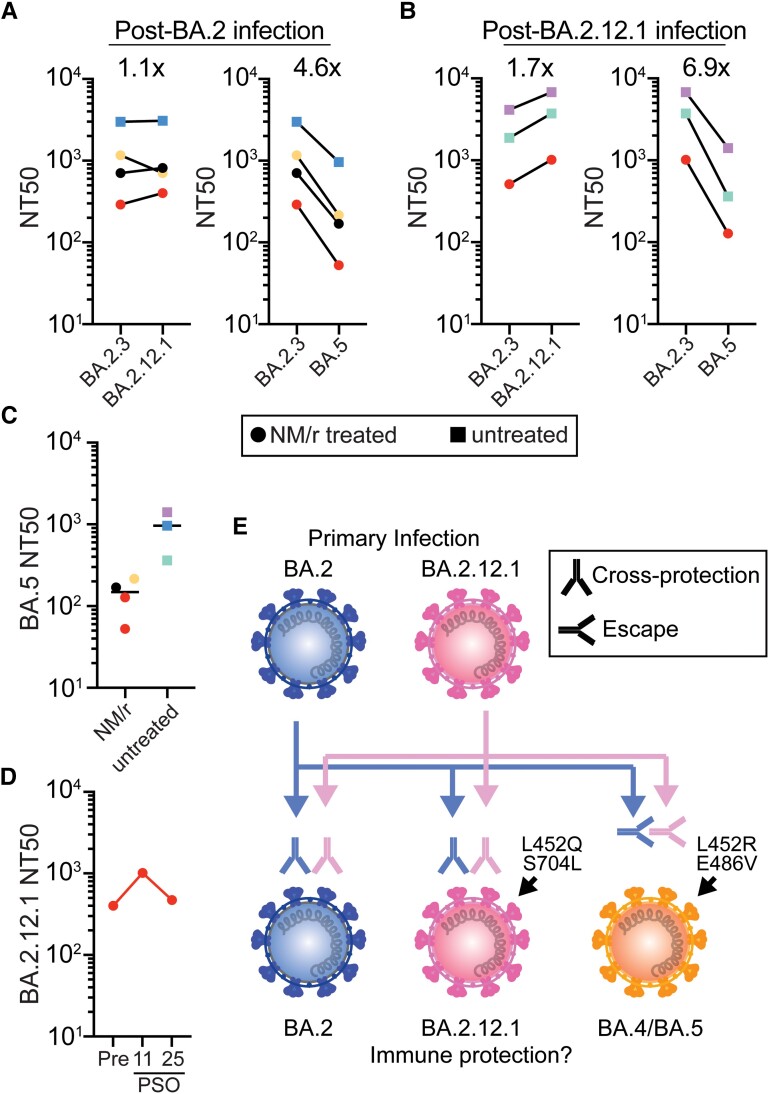
Comparison of neutralization against authentic severe acute respiratory syndrome coronavirus 2 Omicron subvariants after infection with BA.2 or BA.2.12.1. (A–D) The geometric mean NT50s are shown and were calculated from at least 2 independent experiments with 2 biological replicates. (A and B) the NT50s for each individual are connected with a line. The factor of difference between subvariants for each individual was calculated and the average indicated. (C) BA.5 NT50 in untreated and nirmatrelvir/ritonavir (NM/r) treated individuals with median indicated (D) BA.2.12.1 NT50s in a single NM/r treated individual over time. Preinfection (Pre) or postinfection, day 11 or 25 postsymptom onset (PSO), NT50s are shown. (E) Graphical abstract of subvariant cross-protection or escape after infection.

We evaluated persons who had been vaccinated and boosted with messenger ribonucleic acid vaccines and infected with BA.2 or BA.2.12.1 a median of 16 days earlier (range, 11 to 63 days) (Supplementary Table 1) [10]. We then measured NAb titers against authentic SARS-CoV-2 Omicron virus subvariants BA.2.3 (which has an identical spike protein to BA.2), BA.2.12.1, and BA.5 after 4 BA.2 and 3 BA.2.12.1 infections. Individuals infected with BA.2 or BA.2.12.1 had similar neutralization titers against BA.2 and BA.2.12.1 (<1.8-fold difference) (Figure 1*A* and *B*), whereas BA.5 escaped neutralization by both groups. On average, neutralization for BA.5 was 4.6-fold lower than BA.2 in individuals previously infected with BA.2 and 6.9-fold lower than BA.2.12.1 in those previously infected with BA.2.12.1 (Figure 1*A* and *B*).

To assess whether NM/r treatment impacted NAb responses, we evaluated persons who were and were not treated with NM/r during their infections. All treated individuals started NM/r by day 3 of symptoms (Supplementary Table 1). Individuals treated with NM/r (circles) had lower NAb levels against Omicron subvariants, including BA.5, than those who were untreated (squares) (Figure 1*A*–*C*). One individual with previous BA.2 infection experienced reinfection with BA.2.12.1 76 days after initial infection (Figure 1*A*–*C*, red circles). Both infections were treated with NM/r starting on day 2 of symptoms. Upon reinfection and retreatment, neutralization against BA.2.12.1 increased 2.5-fold (from 401 to 1012) at 11 days postsymptom onset (PSO) but was declining by 25 days PSO (NT50 596) (Figure 1*D*).

## DISCUSSION

These data show that NAb responses in vaccinated individuals infected with BA.2 or BA.2.12.1, which harbors an L452Q spike mutation, show similar cross-neutralization, but these NAb responses do not neutralize BA.4 and BA.5 variants, which contain L452R and F486V mutations (Figure 1*E*). In addition, persons treated with NM/r during their infections had lower NAb titers than untreated individuals, and 1 person with previous BA.2 infection treated with NM/r had lower NAb titers and then experienced rapid reinfection with BA.2.12.1.

## CONCLUSIONS

Although limited by a small sample size, our data provide preliminary evidence that early treatment with NM/r may limit the development of a SARS-CoV-2 NAb responses. This phenomena should be investigated in larger studies. Taken together, these data may help explain the rapid emergence of BA.4 and BA.5 subvariants in populations that experienced recent surges of BA.2 and BA.2.12.1 infections, and although early antiviral treatment prevents severe SARS-CoV-2 disease, it does not obviate the need for subsequent vaccination or boosters to promote protective immune responses.

## Supplementary Material

ofad154_Supplementary_Data

## Supplementary Data

Supplementary materials are available at *Open Forum Infectious Diseases* online. Consisting of data provided by the authors to benefit the reader, the posted materials are not copyedited and are the sole responsibility of the authors, so questions or comments should be addressed to the corresponding author.
