# Supplementary material for: Neutralizing Antibody Responses After Severe Acute Respiratory Syndrome Coronavirus 2 BA.2 and BA.2.12.1 Infection Do Not Neutralize BA.4 and BA.5 and Can Be Blunted by Nirmatrelvir/Ritonavir Treatment
Source: Open Forum Infect Dis. 2023 Mar 21;10(4):ofad154. doi: 10.1093/ofid/ofad154 (PMC10122487; doi:10.1093/ofid/ofad154)
Supplement: ofad154_Supplementary_Data [file ofad154_supplementary_data.docx]

**Supplemental Information**

Title: Neutralizing Antibody Responses After SARS-CoV-2 BA.2 and BA.2.12.1 Infection Do Not Neutralize BA.4 and BA.5 and Can Be Blunted By Nirmatrelvir/Ritonavir (NM/r) Treatment

**Supplementary Table 1**

|  | Vaccine 1 & 2 | Booster/s | BA.2 SO or +test | BA.2.12.1 SO or +test | NM/r treated | Days from SO or +test to sampling |
| --- | --- | --- | --- | --- | --- | --- |
| BA.2 | BNT162b2 12/22/2020  01/14/2021 | BNT162b2 09/27/2021 | 4/11/22 | NA | No | 23 |
| BA.2 | BNT162b2 05/10/2021  06/02/2021 | BNT162b2 02/09/2022 | 4/14/22 | NA | Yes | 63 |
| BA.2 | BNT162b2  03/01/2021  03/22/2021 | mRNA-1273 12/20/2021 | 4/13/22 | NA | Yes | 15 |
| BA.2  and BA.2.12.1 | BNT162b2 03/01/2021  03/22/2021 | BNT162b2 11/06/2021 | 4/15/22 | 7/1/22 | Yes (BA.2)  Yes (BA.2.12.1) | 61 (BA.2)  11 (BA.2.12.1)  25 (BA.2.12.1) |
| BA.2.12.1 | mRNA-1273 12/31/2020  01/27/2021 | mRNA-1273 11/05/2021  5/27/2022 | NA | 6/29/22 | No | 16 |
| BA.2.12.1 | mRNA-1273 03/20/2021  04/17/2021 | mRNA-1273 11/27/2021 | NA | 6/29/22 | No | 16 |

Vaccination, infection and collection history. BNT162b2 (Pfizer–BioNTech), mRNA-1273 (Moderna), symptom onset (SO), +test (positive COVID-19 test), NM/r (nirmatrelvir/ritonavir, Paxlovid™)
